# Impact of shade in beef feedyards on performance, ear temperature, and heat stress measures

**DOI:** 10.1093/jas/skad004

**Published:** 2023-05-22

**Authors:** Thomas M Winders, Brett A Melton, Bradley M Boyd, Casey N Macken, Andrea K Watson, James C MacDonald, Galen E Erickson

**Affiliations:** Department of Animal Science, University of Nebraska, Lincoln, NE 68583-0908, USA; Department of Animal Science, University of Nebraska, Lincoln, NE 68583-0908, USA; Department of Animal Science, University of Nebraska, Lincoln, NE 68583-0908, USA; Performance Plus Liquids, Inc., Grand Island, NE 68801, USA; Department of Animal Science, University of Nebraska, Lincoln, NE 68583-0908, USA; Department of Animal Science, University of Nebraska, Lincoln, NE 68583-0908, USA; Department of Animal Science, University of Nebraska, Lincoln, NE 68583-0908, USA

**Keywords:** cattle, feedlot, heat, performance, shade, stress

## Abstract

A 2-yr study (year 1: March to September 2017; year 2: February to August 2018) was conducted using crossbred steers (year 1: *n* = 1677; initial body weight [BW] = 372 kg, SD = 47; year 2: *n* = 1713; initial BW = 379 kg, SD = 10) in a commercial feedyard study in Eastern NE to determine the effects of shade on cattle performance, ear temperature, and cattle activity. Two treatments were evaluated using a randomized complete block design (*n* = 5 blocks based on arrival). Treatments were assigned randomly to pens and consisted of five pens without shade (NO SHADE) and five pens with shade (SHADE). Ear temperatures were collected throughout the trials using biometric sensing ear tags on a subset of cattle. Panting scores were collected using a 5 point scale determined visually based on the level of panting occurring on the same subset of steers a minimum of twice weekly from June 8 to August 21 in year 1 and May 29 to July 24 in year 2 by one trained individual each year. In year 1, no differences (*P* ≥ 0.24) were observed for growth performance or carcass characteristics. Dry matter intake (DMI) and average daily gain (ADG) were greater (*P* ≤ 0.04) for SHADE cattle in year 2. Over the entire feeding period in year 1, greater (*P *< 0.01) ear temperature was observed for NO SHADE cattle, but cattle movement was not different (*P* = 0.38) between treatments. When evaluating the entire feeding period in year 2, cattle movement and ear temperature were not different (*P *≥ 0.80) between treatments. Cattle in the SHADE treatment had lower (*P* ≤ 0.04) panting scores in years 1 and 2. These data suggest that providing shade can lessen the negative influence of heat events on DMI and was an effective way to reduce heat stress in feedlot operations, but only impacted ADG if heat events were close to the cattle slaughter date.

## Teaser

When cattle were exposed to heat stress during finishing, dry matter intake, and average daily gain were reduced when stress occurred close to marketing date, but not when marketed after a recovery period, as determined over two consecutive years. Panting, ear temperature, and pen surface temperatures were improved by providing shade, which may improve cattle growth performance.

## Introduction

Livestock have thermal comfort zones that are dependent on a multitude of factors (humidity, air movement, physiological factors), and when the environment is outside the range of the thermal comfort zone, it can result in less-efficient animal production and an economic loss (Mount, 1974). Heat stress is when energy leaving the animal is less than the heat energy produced by that animal, taking the animal out of the thermal comfort zone. Heat stress costs the beef industry an estimated $370 million annually in production losses ranging from decreases in gain or potentially increased death loss (St-Pierre et al., 2003). With potential for reduced performance paired with consumer concerns regarding animal welfare, improving cattle comfort should be considered. Providing shade to cattle in feedyards will decrease solar radiation experienced by the cattle and reduce ground temperature, but will have little to no effect on ambient air temperature (Morrison, 1983). Producers value shade and sprinkler systems for cattle as heat abatement strategies, with 63% of producers deeming shade as the most desirable mitigation strategy (Rusche et al., 2021). Although heat stress effect on cattle is not a novel concept, determining the impact of heat stress abatement on productivity and behavior is needed under commercial conditions and natural exposure. Stressed cattle show visual signs of distress such as an increase in respiration rates and visible panting (Mader et al., 2006). While an increase in performance (Mitlohner et al., 2002; Sullivan et al., 2011) and a decrease in risk of death (Busby and Loy, 1996) have been observed as a result of shade, the data are inconsistent (Mader et al., 1999). The objective of this 2-yr study was to determine the effect of shade on growth performance, ear temperature, and activity in finishing cattle. These trials were designed similarly, with an exception of intentionally starting and slaughtering the year-2 cattle earlier (while maintaining similar days on feed) to avoid a cool period prior to slaughter.

## Materials and Methods

All animal care and management practices were approved by the University of Nebraska-Lincoln Institutional Animal Care and Use Committee (approval number 1478).

### Year 1

Crossbred steers (*n* = 1677; initial body weight [BW] = 372 kg, SD = 47) were utilized that were *Bos Taurus* breeds consisting of primarily British crossbred steers originating from Nebraska auction markets. Cattle were received from March 17 to April 21. Upon arrival, cattle were vaccinated against *infectious bovine rhinotracheitis*, bovine viral diarrhea types 1 and 2, parainfluenza type 3 and bovine respiratory syncytial virus (Titanium 5; Elanco Animal Health, Greenfield, IN), injected with 1% ivermectin and 10% clorsulon solution for gastrointestinal roundworms, lungworms, adult flukes, cattle grubs, suckling lice, and sarcoptic mange mites (Ivermax Plus; Aspen Veterinary Resources; Greeley, CO), poured with 5 mg/mL ivermectin solution for gastrointestinal roundworms, lungworms, cattle grubs, horn flies, suckling and biting lice, and sarcoptic mange mites (Ivermax Pour On; Aspen Veterinary Resources), and implanted with 100 mg trenbolone acetate and 14 mg estradiol benzoate (Synovex Choice; Zoetis; Parsippany, NJ). Cattle were assigned to treatment as they exited the chute by switching a sort gate every third animal.

Cattle were fed two different common diets during the trial due to corn silage supply. Diet one (Start—July 2; DM basis) consisted of 35% dry-rolled corn (DRC), 37% modified distillers grains plus solubles (MDGS), 10% wet corn gluten feed (WCGF), 12% corn silage, 2% corn stalks, and 4% liquid supplement containing 32.4 mg/kg monensin (Rumensin-90, Elanco Animal Health) and 8.5 mg/kg tylosin (Tylan, Elanco Animal Health). Diet two (July 3—Finish; DM basis) consisted of 41% DRC, 41% MDGS, 11% WCGF, 3% corn stalks, and 5% liquid supplement containing 40.5 mg/kg monensin (Rumensin-90, Elanco Animal Health) and 10.7 mg/kg tylosin (Tylan, Elanco Animal Health). Cattle were stepped up to the finishing ration over 21 days (3 steps; diets not shown). Bunk space was provided at 0.31 to 0.34 linear m per steer. Cattle were re-implanted with 200 mg trenbolone acetate and 20 mg estradiol (Component TE-200; Elanco Animal Health) from June 7 to June 27. Cattle hide color distribution was 70% black, 26% red, and 4% white. Cattle were fed for an average of 165 days.

Cattle were blocked by arrival date and shipped by block, with the first block shipping on September 8, and the final block shipping on September 20. Pen live weights were collected prior to slaughter using a truck scale, where trucks were weighed before and after loading cattle. Live weight was assumed to be the difference in weight, divided by number of cattle, and shrunk by 4%. Cattle were slaughtered at Cargill Meat Solutions (Schuyler, NE). Hot carcass weight (HCW) was collected at time of slaughter. Longissimus muscle area, 12th rib fat thickness, and marbling score were collected following a 32 to 34-hr chill. All carcass data were collected and provided by the packing plant. Yield grade was calculated using the equation of YG, where YG = 2.50 + (6.35 × 12th rib fat depth, cm) − (2.06 × LM area, cm^2^) + (0.2 × KPH, %) + (0.0017 × HCW, kg) (Boggs and Merkel, 1993).

Ten pens were assigned randomly to treatment as having shade (SHADE) or no shade (NO SHADE) provided in the pens, with five pens per treatment. Six of the pens were 61 × 122 m and four of the pens were 41 × 122 m. Each pen provided 38 m^2^/steer, large shaded pens provided 3 m^2^/steer of shade while small shaded pens provided 4 m^2^/steer of shade. Shades were all the same size and composed of high-density polyethylene monofilament (NetPro; Stanthorpe Qld, Australia) that excludes 70% of sunlight. This shade structure has been shown to be effective at reducing heat load (Sullivan et al., 2011) and 70% exclusion appears to be effective based on black globe measurements and modeled impact on heat load for cattle (Eigenberg et al., 2010). Cables that run the length and width of pen held the shade 5.5 m above pen surface.

A subset of 20 (small pens) or 30 (large pens) steers from each pen were selected randomly based on processing order prior to receiving the cattle using excel random number generator, and given a Quantified Ag biometric sensing ear tag (Quantified Ag, Lincoln, NE), which has been shown to work for ear temperature and cattle movement data collection (proprietary company data). The tag recorded movement every hour and ear temperature 5 times per hour. Movement does not have a unit associated with it, and temperature was obtained from an infrared reader aimed down the inner ear canal of the animal. Panting scores were obtained by one trained technician on the same subset of animals that had the biometric sensing ear tag at least twice every week from June 8 to August 24 between 1300 and 1700 hr. Panting scores were based on a score of 0 to 4 in 0.5 increments with a score of 0 = no panting and 4.0 = open mouth with tongue fully extended, excessive drooling, and neck extended (Mader et al., 2006).

**Figure 1.**
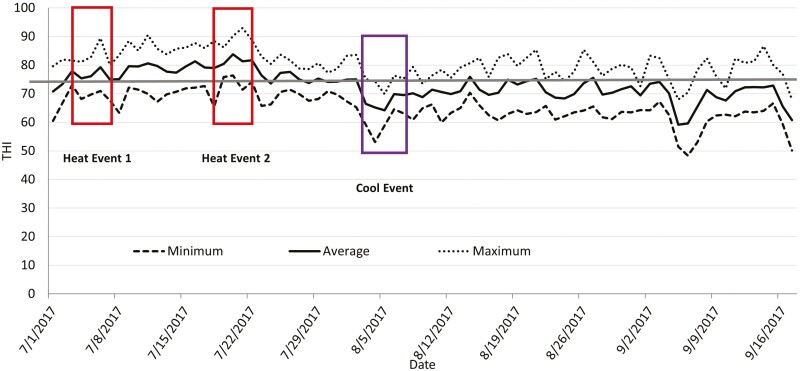
Minimum, maximum, and average adjusted temperature–humidity index (THI) across all days of the trial for year 1. The straight solid line represents the threshold for cattle (THI = 74) set by Livestock Weather Safety Index (LWSI; LCI, 1970).

After the trial, two heat events were defined according to adjusted temperature–humidity index (adjusted THI). Weather data were collected at a weather station located one mile south of the location of the study. Solar radiation was assumed to be a constant 250 W/m^2^ (Mader et al., 2010) in the adjusted THI calculation (weather station did not record these data). A THI of 74 is considered to be a threshold for cattle heat stress based on the Livestock Weather Safety Index (NOAA, 1976), so for this trial the first 5 d that had a daily average adjusted THI greater than 75 was considered heat event one (Event 1; July 3 to July 7). Event two (Event 2; July 18 to July 22) was five consecutive days of the greatest daily average adjusted THI across the feeding period. A cool event was defined as the first five consecutive days following Event 2 that had an average daily adjusted THI below 70. The cool event was from August 3 to August 7. Figure 1 shows the maximum, minimum, and average adjusted THI as well as the weather events previously described. Six temperature and humidity recording devices (Kestral DROP; KestralMeter.com; Minneapolis, MN) were placed in two blocks of pens. One device was placed in NO SHADE pen, and two were placed in SHADE pen (one under the shade, one in the non-shaded area of shaded pen). The devices were hung by wire approximately 3 m off the ground. Ground temperature was recorded with an infrared gun (Extech; Nashua, NH) in each pen on 5 separate days between 1300 and 1600 hr. Temperatures were recorded in 10 separate locations per pen, with 5 of those being under the shaded portion in SHADE pens.

### Statistical analysis

Data were analyzed as a randomized complete block with two treatments and arrival date used as the blocking effect (*n* = 5). Carcass-adjusted performance and carcass characteristics were analyzed using the MIXED procedure of SAS (SAS Institute Inc. Cary, NC) with pen as the experimental unit. Block was included as a fixed effect. Panting scores and biometric ear tag data were analyzed using the MIXED procedure of SAS with pen as the experimental unit and block as a fixed effect. Biometric sensing ear tag data were analyzed for treatment by hour interactions (data were compiled into hourly averages across days). Hour was the repeated variable for biometric sensing ear tag data, and day was the repeated variable for panting score data. Weather data collected to compare between shaded and non-shaded pens were analyzed as repeated measures, with day being the repeated variable. Hour was averaged across days to get one data point per hour. For all repeated measures using hour, compound symmetry was chosen for covariate structure whereas auto-regressive covariate structure was used with repeated day for panting scores. Treatment differences were analyzed as a F-test statistic and considered significant at *P* ≤ 0.05 and trends are discussed with *P *≤ 0.10. Treatment by hour interactions were considered significant at *P *≤ 0.10.

### Year 2

Crossbred steers (*n* = 1713; initial BW = 379 kg, SD = 10) that were *B. Taurus* breeds consisting of primarily British crossbred steers originating from Nebraska auction markets were utilized at the same commercial feedyard in Eastern NE as year 1 to determine the effects of shade on cattle performance, panting scores, ear temperature, and cattle activity. Receiving cattle took place from February 19 to March 5. The same protocol described for year 1 was used for receiving and sorting cattle. Cattle were fed a common diet during the trial consisting of 63% DRC, 20% MDGS, 8% corn cobs, 5% WCGF, and 5% supplement containing 40.5 mg/kg monensin (Rumensin-90, Elanco Animal Health) and 10.7 mg/kg tylosin (Tylan, Elanco Animal Health). Cattle were re-implanted (Synovex Choice; Zoetis; Parsippany, NJ) from May 3 to May 31 depending on receiving date. Cattle hide color distribution was 92% black, 6% red, and 2% white. Cattle were fed for an average of 161 days.

The first block of cattle was shipped on July 25 and the final block was shipped on August 27. Live weight and carcass characteristics data were collected the same as described for year 1. The same treatments (SHADE vs. NO SHADE) were utilized, with the same pen set up with five pens per treatment as previously described.

A subset of 30 steers from each pen were selected to receive the same tag (Quantified Ag biometric sensing ear tag; Quantified Ag) using methods described for year 1. One trained technician recorded panting scores on the same subset of animals that had the biometric sensing ear tag at least twice every week from May 29 to July 24 between 1300 and 1700 hr, and scores were gathered using the same scale as described for year 1.

**Figure 2.**
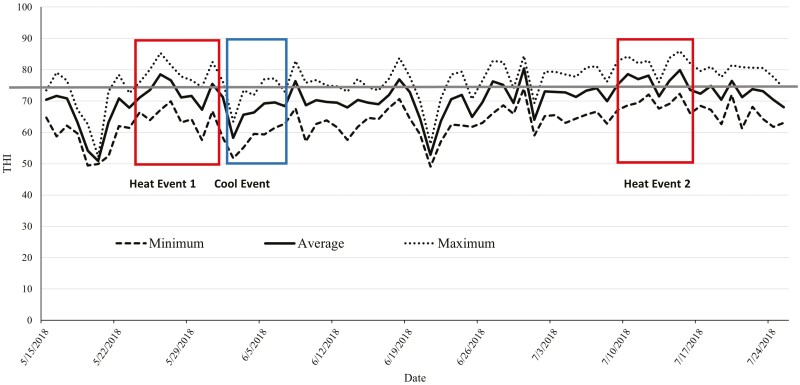
Minimum, maximum, and average adjusted temperature–humidity index (THI) across all days of the trial for year 2. The straight solid line represents the threshold for cattle (THI = 74) set by Livestock Weather Safety Index (LWSI; LCI, 1970).

The adjusted THI values came from a weather station located at the feedyard (Kestral 5400 AG Cattle Heat Stress Tracker; KestralMeter.com; Minneapolis, MN). Heat event 1 (Event 1) was from May 24 to June 1, and heat event 2 (Event 2) was from July 9 to July 16. Both events had a maximum THI greater than 74 each day, with multiple days being greater than 80. The cool event was from June 2 to June 7 and was the first five consecutive days following a heat event with an average daily adjusted THI less than 74. Figure 2 shows the maximum, minimum, and average adjusted THI as well as the weather events previously described. Similar methods described for year 1 for collecting temperature and humidity under the shades and in open pens as well as ground temperature readings were used in year 2.

All cattle performance measures and carcass data were collected similar to year 1.

### Statistical analysis

Data were analyzed as a randomized complete block with two treatments and arrival date used as the blocking effect (*n* = 5). Carcass characteristics, cattle performance, panting scores, and biometric ear tag data were analyzed using the MIXED procedure of SAS (SAS Institute Inc. Cary, NC) with pen as the experimental unit and block included as a fixed effect. Panting scores and biometric sensing ear tag data were analyzed as repeated measures, and biometric sensing ear tag data were tested by pen for treatment by hour interactions (data were compiled into hourly averages). Hour was the repeated variable for biometric sensing ear tag data, and day was the repeated variable for panting score data. Data analysis was identical to year 1. Differences were considered significant at *P* ≤ 0.05 and trends are discussed with *P *≤ 0.10. Treatment by hour interactions was considered significant at *P *≤ 0.10.

## Results

### Year 1

**Figure 3.**
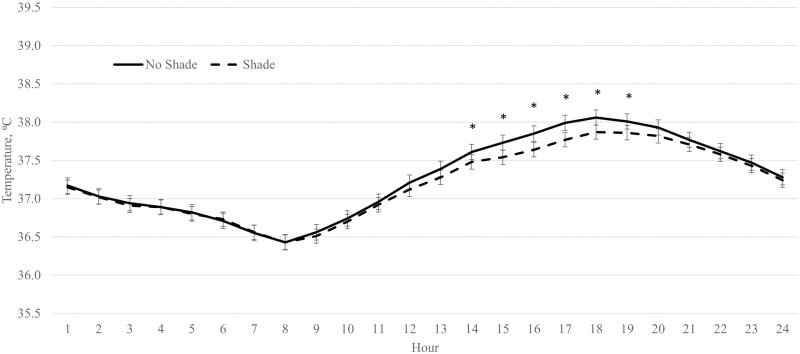
Effect of treatment (SHADE or NO SHADE) on ear temperature of cattle during year 1. Ear temperature was measured 5 times per hour using a biometric sense tag (Quantified Ag, Lincoln, NE). The interaction between treatment and hour was significant (*P* < 0.01). Hourly data averaged across days to get one data point per hour. Treatment difference within hour are significant (*P* < 0.05) at time points denoted with an *.

There were no differences (*P* ≥ 0.24) between SHADE cattle and NO SHADE cattle for dry matter intake (DMI), ADG, feed efficiency (G:F), or carcass characteristics across the entire feeding period (Table 1). Panting scores were reduced (*P* < 0.01) for SHADE cattle compared to NO SHADE cattle across the feeding period (Table 2). Cattle movement was not different (*P* = 0.38) between treatments (Table 2). There was a treatment by hour interaction (*P* < 0.01) for ear temperature. Cattle fed in pens with NO SHADE had greater temperatures from 1300 to 1800 hr compared to cattle fed in pens with SHADE, but ear temperatures were similar all other hours (Figure 3).

**Table 1.** No shade vs. Shade performance and carcass traits (year 1)

	Treatments^1^			
Item	No Shade	Shade	SEM	*P*-value
Performance				
Initial BW, kg	372	372	1	0.75
Adjusted Final BW^2^, kg	668	670	2	0.42
DMI, kg/d	11.1	11.3	0.07	0.31
ADG, kg	1.74	1.76	0.01	0.29
G:F	0.156	0.156	0.001	0.85
Carcass				
HCW^3^, kg	421	423	1.5	0.46
LM area^4^, cm^2^	92.3	93.5	0.7	0.24
12th rib fat, cm	1.51	1.56	0.02	0.49
Marbling^5^	478	479	5	0.92
Calculated YG^6^	3.42	3.43	0.05	0.92

^1^Treatments consisted of five open pens (No Shade) and five shaded (Shade) pens to provide 30 to 45 ft^2^ per animal.

^2^Adjusted final body weight (BW) calculated from hot carcass weight (HCW) using a common 63% dressing percent.

^3^Hot carcass weight.

^4^LM area = longissimus muscle (ribeye) area.

^5^Marbling score: 300 = slight, 400 = small, 500 = modest, etc.

^6^Calculated Yield Grade (YG) = 2.50 + (6.35 × 12th rib fat depth, cm) − (2.06 × LM area, cm^2^) + (0.2 × KPH, %) + (0.0017 × HCW, kg) (Boggs and Merkel, 1993)

**Table 2.** Main effect of treatment on DMI, panting score, movement, and temperature during weather events (year 1)

	Treatment				*P*-value	
Item	No Shade	Shade	SEM	Trt	Hour	Trt*Hour
Total Trial^1^						
Movement	29,032	29,827	636	0.38	<0.01	0.99
Panting Score^2^	0.74	0.55	0.02	<0.01	—	—
Heat Event 1^3^						
Panting Score	0.70	0.27	0.06	<0.01	—	—
Ear Temperature, ºC^4^	38.1	38.0	0.1	0.24	<0.01	0.50
DMI, kg	12.0	12.1	0.2	0.32	—	—
Heat Event 2^5^						
Panting Score	1.76	1.45	0.05	<0.01	—	—
Ear Temperature, ºC	38.2	38.0	0.1	<0.01	<0.01	0.28
DMI, kg	9.5	10.1	0.2	<0.01	—	—
Cool Event^6^						
Movement	30,248	30,593	1595	0.76	<0.01	0.96
Ear Temperature, ºC	36.7	36.5	0.1	0.11	<0.01	0.99
DMI, kg	11.7	12.0	0.1	<0.01	—	—

^1^April 29 to —September 8. 10 pens utilized for all data.

^2^Panting scores were based on a score of 0 to 4 in 0.5 increments.

^3^July 3 to July 7.

^4^Ear temperature was measured using a biometric sense tag (Quantified Ag, Lincoln, NE).

^5^July 18 to July 22.

^6^August 3 to August 7.

**Figure 4.**
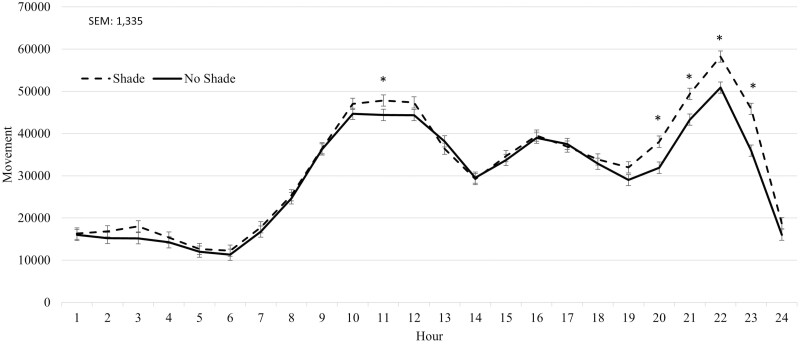
Effect of treatment (SHADE or NO SHADE) on movement of cattle during Heat Event 1 (July 3 to July 7) in year 1. Movement was measured using a biometric sense tag (Quantified Ag, Lincoln, NE) that measured total movement. The interaction between treatment and hour was significant (*P* < 0.01). Hourly data averaged across days to get one data point per hour. Treatment difference within hour are significant (*P* < 0.05) at time points denoted with an *.

**Figure 5.**
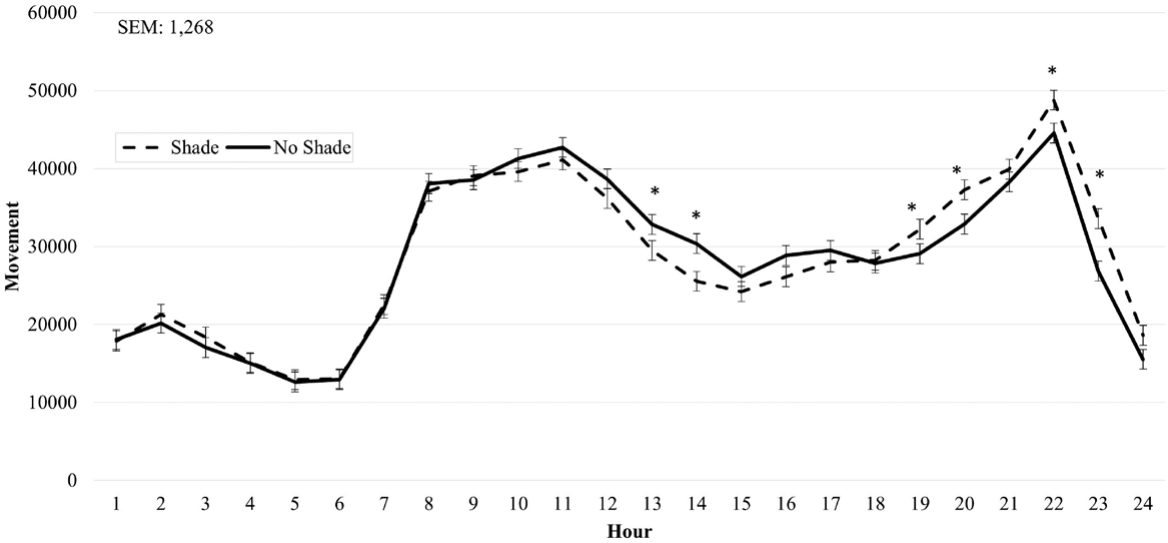
Effect of treatment (SHADE or NO SHADE) on movement of cattle during Heat Event 2 (July 18 to July 22) in year 1. Movement was measured using a biometric sense tag (Quantified Ag, Lincoln, NE) that measured total movement. The interaction between treatment and hour was significant (*P* < 0.01). Hourly data averaged across days to get one data point per hour. Treatment difference within hour are significant (*P* < 0.05) at time points denoted with an *.

During Heat Event 1, SHADE cattle had reduced (*P* < 0.01) panting scores compared to NO SHADE cattle (Table 2). A treatment by hour interaction (*P* < 0.01) was observed for movement. Cattle in the SHADE treatment had greater (*P *≤ 0.05) movement during hours 11 and 20 to 23 (Figure 4), otherwise movement was similar between the two treatments. During Heat Event 2, SHADE cattle had greater (*P* < 0.01) DMI and reduced panting scores compared to NO SHADE cattle (Table 2). A treatment by hour interaction (*P* ≤ 0.05) was observed for movement, where NO SHADE cattle had slightly greater movement from hours 1300 to 1400, while SHADE cattle had greater movement during 1900, 2000, 2200, and 2300 hr (Figure 5). No treatment by hour interaction was observed for ear temperature for Heat Event 1, Heat Event 2, or the Cool Event (Table 2). Ear temperature was not different (*P* = 0.24) between treatments during Heat Event 1. Ear temperature was increased slightly (*P* < 0.01; 0.2 °C) for cattle in NO SHADE compared to SHADE cattle during Heat Event 2 (Table 2). During the Cool Event, SHADE cattle had greater (*P* < 0.01) DMI, but movement and temperature were not different between treatments. Infrared ground temperatures were lowest (*P* < 0.05) underneath the shade at 26.2 °C, while similar between NO SHADE pens (40.7°C) and the open areas of SHADE pens (38.5 °C).

### Year 2

**Figure 6.**
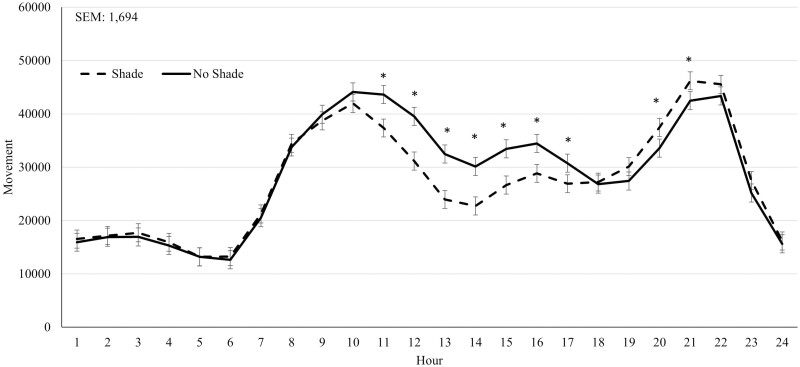
Effect of treatment (SHADE or NO SHADE) on movement of cattle during Heat Event 1 (May 24 to June 1) in year 2. Movement was measured using a biometric sense tag (Quantified Ag, Lincoln, NE) that measured total movement. The interaction between treatment and hour was significant (*P* < 0.01). Hourly data averaged across days to get one data point per hour. Treatment difference within hour are significant (*P* < 0.05) at time points denoted with an *.

**Figure 7.**
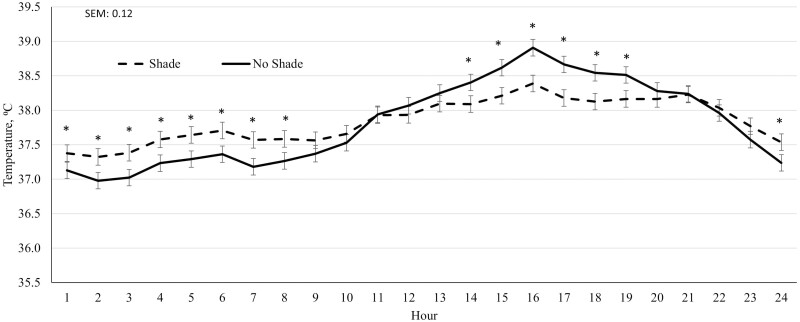
Effect of treatment (SHADE or NO SHADE) on ear temperature of cattle during Heat Event 1 (May 24 to June 1) in year 2. Ear temperature was measured 5 times per hour using a biometric sense tag (Quantified Ag, Lincoln, NE). The interaction between treatment and hour was significant (*P* = 0.06). Hourly data averaged across days to get one data point per hour. Treatment difference within hour are significant (*P* < 0.05) at time points denoted with an *.

**Figure 8.**
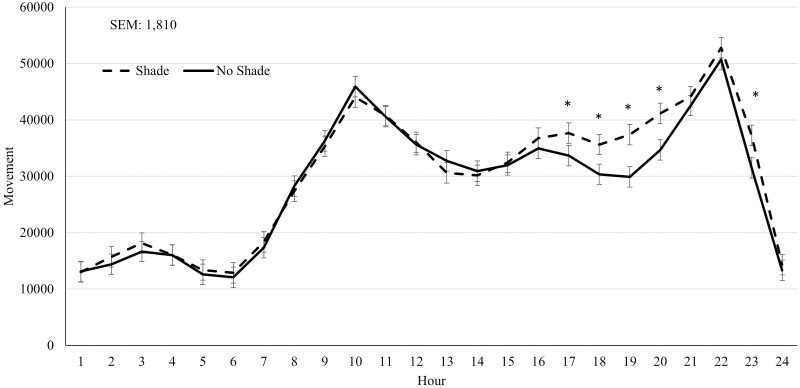
Effect of treatment (SHADE or NO SHADE) on movement of cattle during Heat Event 2 (July 9 to July 16) in year 2. Movement was measured using a biometric sense tag (Quantified Ag, Lincoln, NE) that measured total movement. The interaction between treatment and hour was significant (*P* = 0.06). Hourly data averaged across days to get one data point per hour. Treatment difference within hour are significant (*P* < 0.05)at time points denoted with an *.

**Figure 9.**
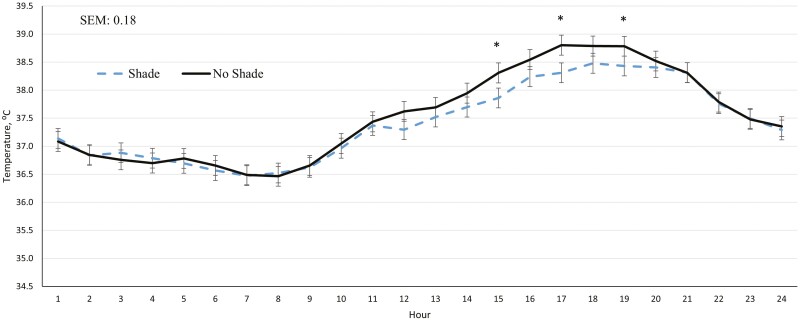
Effect of treatment (SHADE or NO SHADE) on ear temperature of cattle during Heat Event 2 (July 9 to July 16). Ear temperature was measured 5 times per hour using a biometric sense tag (Quantified Ag, Lincoln, NE). The interaction between treatment and hour was significant (*P* = 0.06). Hourly data averaged across days to get one data point per hour. Treatment difference within hour are significant (*P* < 0.05) at time points denoted with an *.

In year 2, cattle on the SHADE treatment had greater (*P* ≤ 0.04) DMI and ADG across the feeding period compared to NO SHADE cattle, while G:F was not influenced (*P* = 0.47) by treatment (Table 3). Longissimus muscle area tended to increase (*P* = 0.06) for SHADE cattle compared to NO SHADE cattle, while final BW and HCW were not significantly different (*P* = 0.11) for SHADE cattle compared to NO SHADE cattle (Table 3). For the entire feeding period in year 2, no interaction between treatment and hour was observed for ear temperature or movement (Table 4). Ear temperature and movement were not different (*P* ≥ 0.80) between treatments across the entire feeding period (Table 4). A treatment by hour interaction (*P* ≤ 0.06) was observed for cattle movement and ear temperature during Heat Event 1 and Heat Event 2. For Heat Event 1, cattle fed in NO SHADE pens had greater movement during hours 1100 to 1700, and SHADE cattle had greater movement during hours 2000 to 2100 (Figure 6) suggesting cattle were moving around more if without shade during the hottest part of the day whereas cattle were not moving as much under shade during the early afternoon hours and then moved slightly more once evening hours and sunset occurred. A treatment by hour interaction (*P* < 0.01) for cattle ear temperature during Heat Event 1 is shown in Figure 7, where SHADE cattle had greater ear temperature during hours 2400 to 0800 while NO SHADE had greater temperature during hours 1400 to 2000. The normal diurnal pattern of body temperature is not as pronounced in cattle that were housed in SHADE pens. In addition, the largest separation of ear temperature between SHADE and NO SHADE cattle was observed in the afternoon hours during Heat Event 2 in year 2. During Heat Event 2 in year 2, SHADE cattle had greater movement during hours 1700 to 2000 plus hour 2300 compared to NO SHADE cattle (Figure 8) whereas movement was similar all other hours of the day during this heat event. Lastly, the treatment by hour interaction observed for cattle ear temperature during Heat Event 2 was due to NO SHADE cattle having greater temperature at hours 1500, 1700, and 1900 compared to SHADE cattle, but similar ear temperatures the remaining hours of the day (Figure 9). Panting scores were greater (*P* ≤ 0.01) for NO SHADE cattle compared to SHADE cattle across the entire feeding period, as well as within both heat events and the cool event (Table 4). DMI was greater (*P* ≤ 0.01) during Heat Event 1, Heat Event 2, and the Cool Event for SHADE compared to NO SHADE (Table 4). Similar to year 1, infrared pen surface temperatures were lowest underneath the shade at 30.3 °C, while not different between NO SHADE pens (43.5 °C) and the open areas of SHADE pens (43.6 °C).

**Table 3.** No shade vs. Shade performance and carcass traits (year 2)

	Treatments^1^			
Item	No Shade	Shade	SEM	*P*-value
Performance				
Initial BW, kg	379	378	1	0.65
Adjusted Final BW^2^, kg	664	671	3	0.11
DMI, kg/d	10.4	10.6	0.01	<0.01
ADG, kg	1.77	1.83	0.01	0.04
G:F	0.170	0.172	0.001	0.47
Carcass				
HCW^3^, kg	418	423	2	0.11
LM area^4^, cm^2^	91.0	94.8	1.3	0.06
12th rib fat, cm	1.50	1.55	0.03	0.32
Marbling^5^	460	459	4	0.87
Calculated YG^6^	3.42	3.31	0.07	0.32

^1^Treatments consisted of five open pens (No Shade) and five shaded (Shade) pens to provide 30 to 45 ft2 per animal.

^2^Adjusted final body weight (BW) calculated from hot carcass weight (HCW) using a common 63% dressing percent.

^3^Hot carcass weight.

^4^LM area = longissimus muscle (ribeye) area.

^5^Marbling score: 300 = slight, 400 = small, 500 = modest, etc.

^6^Calculated Yield Grade (YG) = 2.50 + (6.35 × 12th rib fat depth, cm) − (2.06 × LM area, cm^2^) + (0.2 × KPH, %) + (0.0017 × HCW, kg) (Boggs and Merkel, 1993).

**Table 4.** Main effect of treatment on DMI, panting score, movement, and temperature during weather events (year 2)

Item	Treatment		SEM	Trt	*P*-value	
	No Shade	Shade			Hour	Trt*Hour
Total Trial^1^						
Movement	28,858	28,804	395	0.93	<0.01	0.99
Ear Temperature, ºC^2^	36.6	36.6	0.1	0.80	<0.01	0.31
Panting Score^3^	0.98	0.70	0.02	<0.01	—	—
Heat Event 1^4^						
Panting Score	0.70	0.27	0.06	<0.01	—	—
DMI, kg	9.1	10.9	0.23	<0.01	—	—
Cool Event^5^						
Movement	31,694	31,846	472	0.83	<0.01	0.32
Ear Temperature, ºC	36.8	37.0	0.1	0.08	<0.01	0.27
Panting Score	0.42	0.26	0.04	0.01	—	—
DMI, kg	9.8	10.6	0.05	<0.01	—	—
Heat Event 2^6^						
Panting Score	1.76	1.45	0.05	<0.01	—	—
DMI, kg	10.3	10.6	0.14	0.14	—	—

^1^February 26 to July 25.

^2^Ear temperature was measured using a biometric sense tag (Quantified Ag, Lincoln, NE).

^3^Panting scores were based on a score of 0 to 4 in 0.5 increments.

^4^May 24 to June 1.

^5^June 2 to June 7.

^6^July 9 to July 16.

## Discussion

Although evaluating heat stress effects on cattle is not a novel concept, there are limited commercial trial data available, making these performance data very useful to evaluate productivity of cattle under naturally exposed heat stress events. The effect of shade on cattle performance is inconsistent across trials, likely because of a variety of factors, such as location, weather, cattle type, facilities, and year among others (Mader et al.,1999; Mitlӧhner et al., 2001). Furthermore, dark-colored cattle are likely to bunch more, pant more, have higher tympanic temperatures relative to light-colored cattle and a greater propensity for death from heat stress relative to light-colored cattle (Busby and Loy, 1996; Mader et al., 2002). Shades are commonly used in the feedlot industry (Rusche et al., 2021), although growth performance benefits are inconsistent (Mader et al., 1999; Mitlӧhner et al., 2001). Performance effects are largely a result of a decrease in DMI during heat stress (Morrison, 1983; Hahn, 1995, 1999), however, more recent data suggest that the immune system glucose consumption during heat stress can account for 50% of production losses in dairy cattle (Baumgard and Rhoads, 2013). The present 2-yr trial illustrates the effect that year can have on performance of cattle from providing shade. In year one, no differences in growth performance or carcass characteristics were observed, while in year 2 cattle DMI and ADG were greater for SHADE cattle compared to NO SHADE cattle. Mitlӧhner et al. (2001) fed 77 heifers in a research feedyard in Texas and provided shade for half of the cattle. The shaded cattle had greater DMI, ADG, and final BW. These authors observed a 7% decrease in DMI for cattle without shade compared to shaded cattle. This reduction in DMI is greater than 2% decrease overall, and lower than the 9.5% average reduction in DMI during the heat events observed in year 2 of the current study. Furthermore, the reduction in DMI observed during heat events in year 1 are comparable to the reduction observed by Mitlӧhner et al. (2001), but was only temporary as no difference in DMI was observed overall. Mitlӧhner et al. (2002) fed 168 heifers in a research feedlot in Texas comparing shade to no shade and observed an increase in DMI, ADG, and final BW for shaded cattle relative to non-shaded cattle. Boyd et al. (2015) fed cattle in a Nebraska feedyard in a similar designed study (shade vs. no shade) and observed a tendency for ADG and final BW to be greater for shaded cattle, while DMI and G:F were not influenced. Greater HCW has been observed for shaded cattle compared to non-shaded (Mitlӧhner et al., 2001, 2002), although a change in HCW was not observed (*P* > 0.12) in the present trials. In both years 1 and 2 of the current trial, SHADE cattle had lower panting scores than NO SHADE cattle overall, during heat events, and during cool events. Cattle fed with shade having lower panting scores in the current studies agrees with lower respiration rates observed by Mitlӧhner et al. (2002) for cattle fed in shaded pens compared to open pens. Interestingly, Boyd et al. (2015) did not observe a difference in panting scores between treatments, and Brown-Brandl et al. (2005) only observed increased panting during heat events with no difference during cool events. Defining panting score can vary, and interpretation between trained technicians can also vary, perhaps explaining the differing results across experiments.

Cattle movement observed in both years 1 and 2 of the present trial is similar to that observed by Mitlӧhner et al. (2002). These authors video recorded cattle for 24 hr and categorized movement based off the recordings. They observed cattle with shade have similar movement activity to cattle without shade from hours 0100 to 0700, and from 2200 to 2400. These results are similar to what was observed in both years 1 and 2 of the current study where movement activity is influenced by presence of shades during certain hours of the day. This could be explained by SHADE cattle laying under shade during the greatest heat of the day and moving later in the night when it is cooler. Mitlӧhner et al. (2002) suggest an increase in non-shaded cattle movement between hour 1900 and 2300 can be attributed to agonistic and bulling behavior. This behavior can lead to dark cutters and dust generation. This differs from what was observed in both years 1 and 2 of this trial, where cattle movement from SHADE cattle was greater at times during the night. The movement data collected in the present trial does not specify what the movement is, so concluding agonistic and bulling behavior cannot be confirmed. It could be theorized that SHADE cattle move less during the day to avoid the sunlight and make up for it by more feeding and drinking at night, resulting in greater movement during these hours.

No differences in growth performance or carcass characteristics were observed in year 1, likely because of the later slaughter date (but similar days on feed) as well as cooler weather at the end of the feeding period, potentially allowing for compensatory gain to take place, effectively mitigating any performance differences that occurred during heat events. The idea of compensatory growth following a heat event is supported by Mitlӧhner et al. (2001). In year 2, cattle were slaughtered a month earlier, decreasing the chance of cooler weather allowing for compensatory gain to influence the growth of these cattle.

Across this 2-yr trial, it appears that providing shade can potentially reduced heat stress on cattle, illustrated by reduced panting scores both years. Furthermore, animal movement/behavior is influenced when shade is provided, with SHADE cattle moving less during afternoon hours. Growth performance and carcass characteristic outcomes varied between the 2 yr, but cattle provided shade do not reduce DMI as much which was either temporary like in the first year or observed for the overall intake like in year 2. These data suggests that if slaughter dates are close to heat events, then intake and gain can be increased due to providing shade. These results warrant further investigation into the effects of heat stress severity under natural conditions and the timing of those stress events during the feeding period.

## Abbreviations:

ADG: average daily gain
BW: body weight
DMI: dry matter intake
DRC: dry-rolled corn
G:F: , feed efficiency
HCW: hot carcass weight;
MDGS: , modified distillers grains plus solubles
THI: temperature humidity index
WCGF: wet corn gluten feed
